# Therapeutic drug monitoring and neutralizing anti-drug antibody detection to optimize TNF-alpha inhibitor treatment for uveitis

**DOI:** 10.3389/fopht.2025.1432935

**Published:** 2025-01-28

**Authors:** Howard C. Chen, Jenny Shunyakova, Amit K. Reddy, Srujay Pandiri, Lynn Hassman

**Affiliations:** ^1^ John F. Hardesty Department of Ophthalmology and Visual Sciences, Washington University School of Medicine, St. Louis, MO, United States; ^2^ Department of Ophthalmology, University of Kansas City School of Medicine, Kansas City, MO, United States; ^3^ Department of Ophthalmology, University of Colorado, Aurora, CO, United States

**Keywords:** therapeutic drug monitoring, anti-drug antibodies, TNF-alpha inhibitors, uveitis, adalimumab, infliximab, neutralizing anti-drug antibody

## Abstract

**Background:**

Adalimumab taken every other week is an effective treatment in patients with chronic refractory uveitis. Patients who have a suboptimal response to this treatment may suffer from recurrent inflammation and vision loss. Here, we investigated the use of therapeutic drug monitoring and neutralizing anti-drug antibody detection as a strategy to optimize tumor necrosis factor (TNF)-alpha inhibitor treatment in patients who have a suboptimal response to the initial dosing of adalimumab.

**Method:**

Retrospective cohort study performed in two tertiary referral uveitis services in the United States between 2015 to 2023. Patients with non-infectious uveitis who had a suboptimal response to every two-week dosing of adalimumab and underwent serum adalimumab level with reflex to anti-drug antibody testing were followed. Patients were considered to have neutralizing drug antibodies when serum drug levels were low (less than or equal to 6 mcg/mL) and anti-adalimumab antibodies were present on reflex testing. Treatment adjustment was made by clinicians with the knowledge of serum adalimumab level and the presence or absence of neutralizing drug antibodies. Every two-week dosing of adalimumab was either escalated to weekly dosing or switched to infliximab, an alternate TNF-alpha inhibitor, based on these findings. The primary outcome was success or failure at 12 months, as determined by disease inactivity on steroid-sparing therapy.

**Results:**

32 patients with suboptimal response to the initial dosing of adalimumab were included. 31.2% (n=10) of patients were found to have neutralizing drug antibodies. All patients with neutralizing drug antibodies underwent a medication switch to infliximab with a remission rate of 40% at 12 months. Patients without neutralizing drug antibodies (n=22) underwent dose escalation (77.3%; n=17) or medication switch (22.7%; n=5) and achieved a remission rate of 68.2% at 12 months. Altogether, treatment adjustment based on therapeutic drug monitoring and neutralizing drug antibody detection, in our cohort, resulted in a remission rate of 62.5%.

**Conclusions:**

For patients with uveitis experiencing suboptimal therapeutic response to adalimumab dosed every two weeks, therapeutic drug monitoring and neutralizing drug antibody detection may help clinicians optimize TNF-alpha inhibitor treatment.

## 1 Introduction

Non-infectious uveitis is characterized by inflammation of the intraocular uveal tract. This disease is responsible for 10% of blindness in the United States and disproportionately affects working-aged people, making it a significant cause of vision-related disability (1). Non-infectious uveitis can be classified by anatomic involvement: anterior uveitis, scleritis, intermediate uveitis, posterior uveitis, and pan-uveitis (2). The main causes of vision loss associated with non-infectious uveitis are the development of cystoid macular edema, glaucoma, and cataracts (3).

The primary objective of therapy is to reduce intraocular inflammation to prevent disease progression and restore visual function. Corticosteroids, either local or systemic, are commonly used as the first-line treatment in the acute setting (4). However, prolonged use of local steroids is linked to the development of glaucoma and secondary cataracts (4, 5). Systemic steroids also have side effects including diabetes mellitus, osteoporosis, and increased infection risks. For persistent or severe uveitis, current steroid-sparing therapies focus on targeting specific immunologic pathways. One of these pathways is the tumor-necrosis-factor-alpha (TNF-alpha) pathway. The TNF-alpha pathway is a pro-inflammatory pathway triggered by the binding of TNF-alpha cytokine to the TNF-alpha receptor (6). TNF-alpha inhibitors (TNFi), including adalimumab, etanercept, golimumab, and certolizumab, are biologics that bind to TNF-alpha, block the ligation of TNF receptors, and inhibit its downstream cascade (6, 7).

The FDA approved the use of adalimumab for non-infectious intermediate, posterior, or panuveitis (NIPPU) uveitis in 2016. TNFi are now a mainstay of treatment in patients with chronic refractory uveitis. Adalimumab taken every other week has been shown to lower the risk of uveitis recurrences and visual impairment compared to placebo (8–11). This regimen is effective for approximately 70% of patients with non-infectious uveitis (10, 11). However, a substantial number of patients have a suboptimal response, defined as a partial response or loss of response. One important contributor to suboptimal response is the formation of anti-drug antibodies (ADA), which are antibodies produced by the immune system directed toward the biological drug (12). It is hypothesized that ADA binds to target drugs, causing increased drug clearance and neutralization, thereby leading to reduced drug levels (12). Many studies have shown that the formation of ADA has been associated with decreased serum drug levels, loss of therapeutic response, and higher recurrence rate in the treatment of chronic inflammatory diseases such as inflammatory bowel disease, rheumatoid arthritis, psoriasis, and chronic refractory uveitis (13–21). Studies have also shown that patients who developed ADAs to a certain TNFi have improved responses from switching to another alternative TNFi (22–24).

Therapeutic drug monitoring (TDM) and ADA detection are strategies clinicians use to measure serum drug concentrations to optimize treatment. Most studies evaluating the efficacy of TDM are found in the gastroenterology and rheumatology literature. In chronic inflammatory diseases such as inflammatory bowel disease and rheumatoid arthritis, TDM of adalimumab trough levels to guide treatment adjustments can improve clinical outcomes and be cost-effective, especially in recurrent or persistent inflammation (25, 26). The use of TDM in treating chronic refractory uveitis has not been the standard of practice, and the literature on the efficacy of TDM in this group of patients is limited. Sejournet et al. and Bellur et al. have shown that uveitis patients who are non-responders to adalimumab are significantly more likely to have low serum adalimumab levels and the presence of ADA (17, 18). Sejournet et al. have proposed an algorithm for reactive TDM and ADA detection in patients with suboptimal response to adalimumab similar to the strategy used in gastroenterology, rheumatology, and dermatology (17). The clinical utility and efficacy of this algorithm in chronic refractory uveitis patients have yet to be fully elucidated.

Considering this background, this study sought to 1) examine the efficacy of using TDM and neutralizing ADA detection to guide treatment decisions, 2) compare the remission rates in patients with and without neutralizing drug antibodies in 12 months, and 3) evaluate the prevalence of neutralizing drug antibodies in uveitis patients who have a suboptimal response to the initial dose of adalimumab.

## 2 Methods

### 2.1 Study design

This study involved human research and was approved by the Institutional Review Board at the Human Research Protection Office (HRPO) of Washington University in St. Louis and by the Colorado Multiple Institutional Review Board. All work conformed to the tenets of the Declaration of Helsinki. A retrospective chart review of adult and pediatric patients with non-infectious uveitis who underwent adalimumab drug-level testing due to suboptimal response to initial dosing of adalimumab was conducted between 2015 and 2023. Inclusion criteria were 1) a clinical diagnosis of non-infectious uveitis, 2) suboptimal response to adalimumab 40mg dosed every two weeks, and 3) undergoing serum adalimumab level with reflex to ADA testing. Suboptimal response was determined by a uveitis-trained specialist based on active uveitis according to the Standardization of Uveitis Nomenclature II (SUN II) criteria (27). Patients who were lost to follow-up before 12 months were excluded.

Patients underwent adalimumab drug-level testing by enzyme-linked immunosorbent assay (ELISA). In patients with low adalimumab levels, defined as less than or equal to 6 mcg/mL, reflex testing of ADA was obtained. When adalimumab concentrations are greater than 6 mcg/mL, clinically relevant antibodies to adalimumab are unlikely and reflex testing was not performed. Patients with low adalimumab levels and the presence of ADA are considered to have neutralizing ADA. Patients are presumed to have no neutralizing ADA if they have an adequate adalimumab level, defined by greater than 6 mcg/mL, or a low adalimumab level, but reflex ADA testing is negative. The clinician used serum adalimumab level and the presence or absence of neutralizing ADA to make clinical decisions regarding treatment adjustments.

Charts were reviewed for patient characteristics, including age, sex, and body-mass index (BMI). Uveitis was classified as anterior uveitis, scleritis, and noninfectious intermediate, posterior, or pan-uveitis (NIIPPU). Treatment data were collected, such as duration of treatment, serum drug level, anti-drug antibodies (if reflex testing was done), therapy changes, concomitant immune suppression, use of topical steroids, and use of intraocular or periocular steroid implants.

Patients were followed every three to four months. The end-point was success or failure at 12 months. Whether uveitis was active or inactive was determined by the clinician based on clinical exam, optical coherence tomography, and fluorescein angiography. Success was determined by 1) inactive or minimally active non-infectious anterior, intermediate, posterior, or panuveitis, as defined by SUN II criteria as ≤ 0.5+ anterior chamber cells, ≤ 0.5+ vitreous haze grade, and no active retinal/choroidal lesions for a minimum of 4 weeks, and 2) on steroid-sparing therapy, which was defined by no more than prednisone 7.5 mg daily, topical prednisolone 1% two times daily, topical difluprednate 0.05% once daily, and/or four months or more since last intraocular or periocular steroid implantation.

### 2.2 Statistical analysis

Continuous variables were summarized as median (IQR) and mean and compared using Wilcoxon two-sample test. Discrete variables were compared using the chi-squared test and Fisher’s exact test. All statistical tests were two-tailed. P values less than 0.05 were considered to be statistically significant.

## 3 Results

### 3.1 Baseline patient characteristics

A total of 32 patients with at least 12 months follow-up who either only partially responded or experienced secondary failure to 40mg adalimumab every two weeks underwent serum adalimumab with reflex to ADA measurements. The demographics, characteristics, and outcomes of all the patients are listed in **Table 1** and summarized separately by the presence and absence of neutralizing ADA in **Table 2**.

**Table 1:** Patient demographics, characteristics, and result of therapy.

	Patient Number	Age (years)	Sex	BMI	Diagnosis	Time (years) since initial diagnosis	Time (months) to failure	Concomitant antimetabolite	Drug level (mcg/mL)	Success/failure at 12 months
Neutralizing ADA	1	12	female	16.8	AU	2.9	52.3	yes	4.1	success
	2	49	Male	24.3	NIIPPU	2.2	7.1	yes	1.5	success
	3	40	female	36.7	NIIPPU	6.7	9.9	no	undetectable	success
	4	19	female	18.0	AU	1.0	39.0	no	undetectable	success
	5	41	Female	29.4	S	0.1	4.6	no	undetectable	failure
	6	36	female	38.5	NIIPPU	8.7	3.9	yes	2.4	failure
	7	58	female	42.4	NIIPPU	3.9	38.3	yes	undetectable	failure
	8	39	Male	30.6	NIIPPU	0.4	6.6	yes	1.4	failure
	9	30	Male	30.4	NIIPPU	0.6	7.3	yes	6.0	failure
	10	44	female	31.2	NIIPPU	0.6	9.2	yes	5.0	failure
Without Neutralizing ADA	11	59	Female	22.1	NIIPPU	1.7	14.8	yes	19.6	success
	12	47	female	29.9	NIIPPU	0.9	3.8	yes	9.8	success
	13	37	Male	29.0	NIIPPU	21.0	31.0	yes	23.7	success
	14	39	Female	28.7	AU	9.7	16.3	yes	15.0	failure
	15	35	Male	30.0	AU	11.0	38.0	no	19.1	success
	16	69	Female	24.1	NIIPPU	1.2	3.6	no	19.7	success
	17	32	male	47.4	NIIPPU	0.4	23.0	yes	8.6	success
	18	29	female	28.6	NIIPPU	6.3	16.1	no	9.0	success
	19	76	male	28.9	NIIPPU	0.2	12.0	yes	10.1	success
	20	24	female	28.4	AU	16.5	13.5	yes	20.0	success
	21	62	female	31.3	NIIPPU	14.3	24.1	yes	0.8	success
	22	61	female	40.3	NIIPPU	0.0	15.8	yes	6.8	success
	23	59	female	48.8	NIIPPU	1.3	7.1	yes	6.4	success
	24	33	female	15.6	AU	1.2	46.1	no	18.0	success
	25	22	Male	20.0	NIIPPU	1.0	35.0	yes	23.2	success
	26	36	Male	34.0	NIIPPU	0.0	50.0	yes	11.0	success
	27	69	Female	24.8	NIIPPU	1.2	2.0	no	11.0	success
	28	75	Female	27.1	NIIPPU	5.5	12.2	yes	18.4	failure
	29	71	Female	30.2	NIIPPU	1.2	4.0	yes	13.0	failure
	30	62	female	23.7	NIIPPU	3.3	5.3	yes	22.5	failure
	31	51	female	23.1	NIIPPU	3.3	9.3	yes	10.9	failure
	32	34	female	53.4	NIIPPU	10	18.6	yes	3.9	failure

AU, Anterior Uveitis; S, Scleritis; NIIPPU, Non-Infectious Intermediate, Posterior, or Panuveitis.

**Table 2:** Comparison of predictors for patients without and with neutralizing drug antibodies.

Predictors	With neutralizing ADA (No. = 10)	Without neutralizing ADA (No. = 22)	p-value
Sex, No.:			
Female	7 (70%)	16 (73%)	1.0*
Male	3 (30%)	6 (27%)	
NIPPU Dx, No.:			
No	3 (30%)	4 (18%)	0.65*
Yes	7 (70%)	18 (82%)	
Concomitant therapy, No.:			
No	3 (30%)	5 (23%)	0.68*
Yes	7 (70%)	17 (77%)	
Age, mean ± SD	36.8 ± 13.5	49.2 ± 17.6	0.13†
median (IQR)	39.5 (14.4)	49.0 (28.8)	
min, max	12.2, 57.8	22.0, 76.0	
BMI, mean ± SD	29.8 ± 8.3	30.4 ± 9.4	0.58†
median (IQR)	30.5 (12.3)	28.8 (7.1)	
min, max	16.8, 42.4	15.6, 53.4	

Analysis tests the null hypothesis that the predictor is not significantly different for patients without compared to with neutralizing drug antibodies.

ADA, anti-drug antibodies; IQR, interquartile range; NA, not applicable; No,. number of participants; SD, standard deviation.

*P-value by Fisher’s exact test.

^†^P-value by normal approximation Wilcoxon two-sample test.

The ages in our cohort ranged from 12 to 76 and the mean age was not significantly different between patients with and without neutralizing ADAs. Sex was predominantly female and not significantly different in both groups. NIIPPU was the most common diagnosis of uveitis in both groups. The time to failure of every two-week dosing of adalimumab ranged from 2 months to 52 months and the mean was not statistically different between the two groups. In the neutralizing ADA group, 70.0% of patients were on concomitant antimetabolite therapy, compared to 77.3% in the group without neutralizing ADA. There was no statistically significant difference (p=0.68) in concomitant antimetabolite use between the two groups.

### 3.2 Prevalence of neutralizing antibody in patients with suboptimal response to adalimumab

Of the 32 patients who had a suboptimal response to adalimumab, 10 patients (31.2%) potentially had neutralizing ADAs, defined as low adalimumab levels (less than 8/mL) and positive ADA on reflex testing. The mean serum adalimumab level in patients with neutralizing ADA was 2.0 ± 2.3 mcg/mL.

22 (68.8%) patients were presumed to have no neutralizing ADAs. Of these, only 2 patients had low adalimumab levels (less than or equal to 6 mcg/mL), but reflex ADA testing was negative. All the remaining 20 patients had adequate adalimumab levels therefore reflex ADA testing was not obtained. The mean serum adalimumab level in patients without neutralizing ADA was 13.7 ± 6.6 mcg/mL.

### 3.3 Neutralizing antibodies and therapy changes

Therapy changes (dose escalation or medication switch) were determined by the clinician based on the knowledge of the serum adalimumab level and the presence or absence of neutralizing ADA. All 10 patients who developed neutralizing ADAs underwent medication switch to infliximab. None of the patients with neutralizing ADAs underwent dose escalation in adalimumab.

Of the 22 patients who did not have neutralizing ADA, 17 patients (77.2%) underwent escalation to weekly adalimumab dosing. Five patients (22.7%) switched to infliximab. The reasons for switching included insurance preference (2 patients), flare of another systemic rheumatologic disease on adalimumab (1 patient), and adalimumab failure despite high serum adalimumab levels (2 patients).

### 3.4 Therapy changes and remission rate

Altogether, treatment adjustment based on TDM achieved a success rate of 62.5% in patients who previously had suboptimal response to adalimumab.

**Figure 1:**
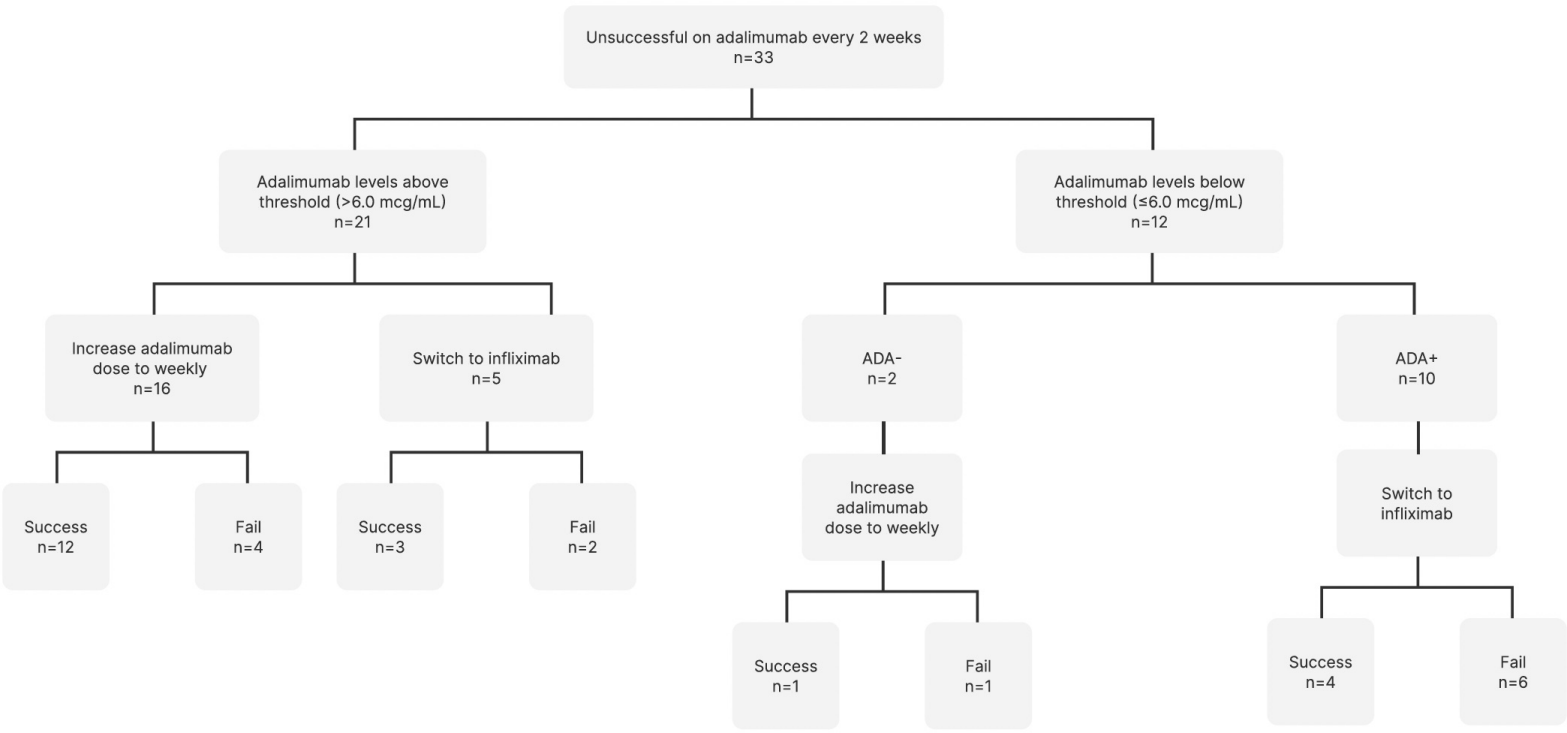
Therapy changes based on therapeutic drug monitoring and anti-drug antibody detection. ADA, Anti-drug antibodies.

Of the 22 patients who did not have neutralizing ADA, 17 patients underwent escalation to weekly adalimumab dosing (**Figure 1**). Of the patients who underwent dose escalation, 12 (70.6%) successfully achieved remission at 12 months, and 5 (29.4%) failed therapy. Five patients switched to infliximab despite adequate adalimumab levels. Of these, three patients (60.0%) succeeded, while two (40.0%) failed.

Of the 10 patients who developed neutralizing ADAs and underwent medication switch to infliximab, four (40.0%) achieved remission, and six (60.0%) failed.

The presence of neutralizing antibodies was not significantly associated with failure/success (p=0.24 by Fisher’s exact test). Of the 13 failures, six (46%) had neutralizing antibodies and seven (54%) did not. Of the 19 successes, four (21%) had neutralizing antibodies and 15 (79%) did not. The odds ratio of treatment success was 3.09 for patients without neutralizing ADA compared to patients with neutralizing ADA, though this was not statistically significant (p = 0.26).

### 3.5 Patient demographics and disease characteristics as predictors for remission rate

Neither sex, age, BMI, NIPPU diagnosis, years since initial diagnosis, nor concomitant immunosuppression had a statistically significant association with remission rates, either in patients with or without neutralizing ADA (**Table 3**).

**Table 3:** Separately for patients without and with neutralizing drug antibodies, a comparison of predictors for patients who failed and did not fail. Analysis tests the null hypothesis that the predictor is not significantly different for patients who failed compared to success.

Predictor	Group = With Neutralizing Drug Antibodies (No. = 10)			Group = Without Neutralizing Drug Antibodies (No. = 22)		
	Failed (No. = 6)	Success (No. = 4)	p-value	Failed (No. = 7)	Success (No. = 15)	p-value
Sex, No.:						
Female	4	3	1.0*	6	10	0.62*
Male	2	1		1	5	
NIPPU Dx, No.:						
No	1	2	0.50*	2	2	0.56*
Yes	5	2		5	13	
Concomitant therapy, No.:						
No	1	2	0.50*	1	4	1.0*
Yes	5	2		6	11	
Age, mean ± SD	41.4 ± 9.4	30.0 ± 17.3	0.46†	52.5 ± 17.2	47.6 ± 18.2	0.40†
median (IQR)	40.2 (8.8)	29.3 (28.8)		51.2 (35.6)	46.8 (29.7)	
min, max	30.1, 57.8	12.2, 49.2		33.5, 75.0	22.0, 76.0	
BMI, mean ± SD	33.8 ± 5.4	23.9 ± 9.1	0.11†	30.9 ± 10.3	30.2 ± 9.3	1.0†
median (IQR)	30.9 (8.1)	21.2 (13.1)		28.7 (6.5)	28.9 (9.9)	
min, max	29.4, 42.4	16.8, 36.7		23.1, 53.4	15.6, 48.8	
Years since initial diagnosis:						0.52†
mean ± SD	2.4 ± 3.4	3.2 ± 2.4	0.34†	6.3 ± 3.9	4.5 ± 6.9 1.2	
median (IQR)	0.6 (3.5)	2.5 (3.2)		5.5 (6.7) 1.2, 11.0	(5.9) 0, 21.0	
min, max	0.1, 8.7	1.0, 6.7				
Dosing:						
Increased Dose	0	0	NA	5	12	1.0*
Switch TNFi	6	4		2	3	

IQR, interquartile range; NA, not applicable; No., number of participants; SD, standard deviation.

*P-value by Fisher’s exact test.

^†^P-value by normal approximation Wilcoxon two-sample test.

## 4 Discussion

### 4.1 Study findings

The formation of ADA to TNFi can neutralize the drug’s ability to block the interaction between TNF and its receptor, reducing the efficacy of the drug, which may lead to partial response or loss of response. ADA can be neutralizing or non-neutralizing. Neutralizing ADA’s are associated with low serum drug levels and are clinically significant (12, 28). In our study, neutralizing ADA was defined by low serum adalimumab levels along with the presence of ADA on reflex testing in patients for whom treatment was ineffective. We found that 31.2% of patients who had suboptimal responses to adalimumab had neutralizing ADA. In comparison, other studies have reported the formation of ADAs ranging from 2.7% to 35.7% (8, 18, 20). Patients with adequate serum adalimumab levels did not undergo reflex ADA testing. Presumably, if ADA were present in these patients, they were either transient or non-neutralizing and therefore clinically insignificant (28, 29).

In our study, clinicians made treatment adjustments based on TDM and the presence of neutralizing ADA. Patients were more likely to undergo dose escalation if they did not have neutralizing ADA. Patients who had neutralizing ADA were transitioned to an alternate TNFi. This is a standard practice pattern employed by gastroenterologists in the treatment of inflammatory bowel disease with biologics (30–32). The rationale is that a threshold serum drug level is required to achieve a therapeutic response and that the presence of neutralizing ADA would bind to the target drug and render any escalation in dose ineffective (12). In our study, infliximab, an alternate TNFi was chosen. Previous studies demonstrated that anti-adalimumab antibodies are restricted to the adalimumab idiotype and do not alter the inhibitory effect of infliximab (12). Our study found that for the 10 patients who developed neutralizing ADAs and underwent medication switch to infliximab, four (40.0%) achieved remission, and six (60.0%) failed. Switching to infliximab may not lead to remission for several reasons including patient demographics and disease characteristics, which we are unable to investigate given the small sample of patients but may be a future area of exploration. Patients may have developed anti-infliximab antibodies. Other steroid-sparing options for patients who have developed ADA and failed infliximab include switching to alternate biologics, intraocular corticosteroids, or combination therapy with other medications such as anti-metabolites.

In patients who had a suboptimal response to the initial every two-week dosing of adalimumab, our study demonstrated that treatment adjustment guided by TDM and neutralizing ADA detection achieved a success rate of 62.5% in 12 months. Another study reported that TDM led to an improvement in response in 87% of uveitis patients who are non-responders (17). In contrast, several retrospective cohort studies evaluating the escalation to weekly adalimumab without TDM found success rates ranging from 56% to 66.6% (33–35). A larger randomized controlled study is needed to determine whether using TDM and neutralizing ADA detection in uveitis improves outcomes.

TDM has already been routinely used to guide treatment in the fields of rheumatology, gastroenterology, and dermatology in patients on TNFi (30–32). Our study further explores the use of TDM as a potential tool in patients with chronic refractory uveitis to help clinicians optimize treatment using TNFi. For patients without neutralizing ADA, we suggest escalating the dose of adalimumab first if the patient can tolerate treatment (33–35). For patients with neutralizing ADA, we recommend switching to an alternative TNFi or biologic.

In patients on TNFi monotherapy, clinicians may also consider adding concomitant immunosuppression therapy such as disease-modifying anti-rheumatic drugs (DMARDs). Although our study did not show a statistically significant difference in concomitant immunosuppression and the presence of neutralizing ADA, some prior studies have shown reduced rates of ADA formation and improved treatment outcomes (20, 37,36, 37). Given prior studies suggesting possible benefits of DMARDs, it might emerge as predictive in larger studies. We also recommend further larger prospective trials on the effects of concomitant immunosuppression therapy.

### 4.2 Limitations and future directions

The current study is limited by its retrospective and observational nature. It is also limited by a smaller sample size, which may limit validity and generalizability. A larger randomized control trial is warranted. Additionally, repeat serum level testing may provide more insight as to whether dose escalation results in higher serum levels and improves clinical response. In patients who were switched to infliximab, measuring serum infliximab levels and detection of neutralizing ANA to infliximab will also help determine whether patients who developed neutralizing ANA to one biologic agent are more likely to develop neutralizing ANA to another.

### 4.3 Conclusion

In summary, TDM and neutralizing ADA detection is a promising strategy to guide treatment modifications in patients who have a suboptimal response to the initial every two-week dosing of adalimumab, a larger prospective randomized trial is warranted.

## Data Availability

The original contributions presented in the study are included in the article/supplementary material. Further inquiries can be directed to the corresponding author.

## References

[1] Darrell RW Wagener HP Kurland LT. Epidemiology of uveitis. Incidence and prevalence in a small urban community. Arch Ophthalmol (Chicago Ill.: 1960). (1962) 68:502–14. doi: 10.1001/archopht.1962.00960030506014 13883604

[2] Heiligenhaus A Rothaus K Pleyer U. Development of classification criteria for uveitis by the standardization of uveitis nomenclature (SUN) working group. Der Ophthalmologe: Z Der Deutschen Ophthalmologischen Gesellschaft. (2021) 118:913–8. doi: 10.1007/s00347-021-01486-2 PMC8413183 34459962

[3] Durrani OM Tehrani NN Marr JE Moradi P Stavrou P Murray PI. Degree, duration, and causes of visual loss in uveitis. Br J Ophthalmol. (2004) 88:1159–62. doi: 10.1136/bjo.2003.037226 PMC1772296 15317708

[4] Gamalero L Simonini G Ferrara G Polizzi S Giani T Cimaz R. Evidence-based treatment for uveitis. Israel Med Assoc Journal: IMAJ. (2019) 21:475–9. 31507124

[5] Jabs DA Rosenbaum JT Foster CS Holland GN Jaffe GJ Louie JS. Guidelines for the use of immunosuppressive drugs in patients with ocular inflammatory disorders: Recommendations of an expert panel. Am J Ophthalmol. (2000) 130:492–513. doi: 10.1016/s0002-9394(00)00659-0 11024423

[6] Jang D-I Lee A-H Shin H-Y Song H-R Park J-H Kang T-B. The role of tumor necrosis factor alpha (TNF-α) in autoimmune disease and current TNF-α Inhibitors in therapeutics. Int J Mol Sci. (2021) 22:2719. doi: 10.3390/ijms22052719 33800290 PMC7962638

[7] Melsheimer R Geldhof A Apaolaza I Schaible T. Remicade^®^ (infliximab): 20 years of contributions to science and medicine. Biologics: Targets Ther. (2019) 13:139–78. doi: 10.2147/BTT.S207246 PMC6679695 31440029

[8] Jaffe GJ Dick AD Brézin AP Nguyen QD Thorne JE Kestelyn P. Adalimumab in patients with active noninfectious uveitis. New Engl J Med. (2016) 375:932–43. doi: 10.1056/NEJMoa1509852 27602665

[9] Suhler EB Jaffe GJ Fortin E Lim LL Merrill PT Dick AD. Long-term safety and efficacy of adalimumab in patients with noninfectious intermediate uveitis, posterior uveitis, or panuveitis. Ophthalmology. (2021) 128:899–909. doi: 10.1016/j.ophtha.2020.10.036 33157077

[10] Suhler EB Adán A Brézin AP Fortin E Goto H Jaffe GJ. Safety and efficacy of adalimumab in patients with noninfectious uveitis in an ongoing open-label study: VISUAL III. Ophthalmology. (2018) 125:1075–87. doi: 10.1016/j.ophtha.2017.12.039 29429764

[11] Suhler EB Lowder CY Goldstein DA Giles T Lauer AK Kurz PA. Adalimumab therapy for refractory uveitis: Results of a multicentre, open-label, prospective trial. Br J Ophthalmol. (2013) 97:481–6. doi: 10.1136/bjophthalmol-2012-302292 23376607

[12] van Schouwenburg PA van de Stadt LA de Jong RN van Buren EEL Kruithof S de Groot E. Adalimumab elicits a restricted anti-idiotypic antibody response in autoimmune patients resulting in functional neutralisation. Ann Rheumatic Dis. (2013) 72:104–9. doi: 10.1136/annrheumdis-2012-201445 22759910

[13] Gorovits B Baltrukonis DJ Bhattacharya I Birchler MA Finco D Sikkema D. Immunoassay methods used in clinical studies for the detection of anti-drug antibodies to adalimumab and infliximab. Clin Exp Immunol. (2018) 192:348–65. doi: 10.1111/cei.13112 PMC5980437 29431871

[14] Wolbink GJ Vis M Lems W Voskuyl AE de Groot E Nurmohamed MT. Development of antiinfliximab antibodies and relationship to clinical response in patients with rheumatoid arthritis. Arthritis Rheumatism. (2006) 54:711–5. doi: 10.1002/art.21671 16508927

[15] Jyssum I Gehin JE Sexton J Kristianslund EK Hu Y Warren DJ. Adalimumab serum levels and anti-drug antibodies: Associations to treatment response and drug survival in inflammatory joint diseases. Rheumatol (Oxford England). (2023) 63(6):kead525. doi: 10.1093/rheumatology/kead525 PMC11147536 37773994

[16] Hinojosa J Muñoz F Martínez-Romero GJ. Relationship between serum adalimumab levels and clinical outcome in the treatment of inflammatory bowel disease. Digestive Dis (Basel Switzerland). (2019) 37:444–50. doi: 10.1159/000499870 31039560

[17] Sejournet L Kerever S Mathis T Kodjikian L Jamilloux Y Seve P. Therapeutic drug monitoring guides the management of patients with chronic non-infectious uveitis treated with adalimumab: A retrospective study. Br J Ophthalmol. (2022) 106:1380–6. doi: 10.1136/bjophthalmol-2021-319072 33875451

[18] Bellur S McHarg M Kongwattananon W Vitale S Sen HN Kodati S. Antidrug antibodies to tumor necrosis factor α Inhibitors in patients with noninfectious uveitis. JAMA Ophthalmol. (2023) 141:150–6. doi: 10.1001/jamaophthalmol.2022.5584 PMC9936342 36547953

[19] Cordero-Coma M Calleja-Antolín S Garzo-García I Nuñez-Garnés AM Álvarez-Castro C Franco-Benito M. Adalimumab for treatment of noninfectious uveitis: immunogenicity and clinical relevance of measuring serum drug levels and antidrug antibodies. Ophthalmology. (2016) 123:2618–25. doi: 10.1016/j.ophtha.2016.08.025 27692527

[20] Eurelings LEM Missotten TOAR van Velthoven MEJ van Daele PLA van Laar JAM van Hagen PM. Long-term follow-up of patients with uveitis treated with adalimumab: response rates and reasons for discontinuation of therapy. Am J Ophthalmol. (2022) 240:194–204. doi: 10.1016/j.ajo.2022.03.017 35314190

[21] Bartelds GM Wijbrandts CA Nurmohamed MT Stapel S Lems WF Aarden L. Clinical response to adalimumab: Relationship to anti-adalimumab antibodies and serum adalimumab concentrations in rheumatoid arthritis. Ann Rheumatic Dis. (2007) 66:921–6. doi: 10.1136/ard.2006.065615 PMC1955110 17301106

[22] Jamnitski A Bartelds GM Nurmohamed MT van Schouwenburg PA van Schaardenburg D Stapel SO. The presence or absence of antibodies to infliximab or adalimumab determines the outcome of switching to etanercept. Ann Rheumatic Dis. (2011) 70:284–8. doi: 10.1136/ard.2010.135111 21068090

[23] Bombardieri S Ruiz AA Fardellone P Geusens P McKenna F Unnebrink K. Effectiveness of adalimumab for rheumatoid arthritis in patients with a history of TNF-antagonist therapy in clinical practice. Rheumatol (Oxford England). (2007) 46:1191–9. doi: 10.1093/rheumatology/kem091 17504821

[24] Rubbert-Roth A Finckh A. Treatment options in patients with rheumatoid arthritis failing initial TNF inhibitor therapy: A critical review. Arthritis Res Ther. (2009) 11 Suppl 1:S1. doi: 10.1186/ar2666 19368701 PMC2669237

[25] Syed N Tolaymat M Brown SA Sivasailam B Cross RK. Proactive drug monitoring is associated with higher persistence to infliximab and adalimumab treatment and lower healthcare utilization compared with reactive and clinical monitoring. Crohn’s Colitis 360. (2020) 2:otaa050. doi: 10.1093/crocol/otaa050 32743546 PMC7380488

[26] Assa A Matar M Turner D Broide E Weiss B Ledder O. Proactive monitoring of adalimumab trough concentration associated with increased clinical remission in children with crohn’s disease compared with reactive monitoring. Gastroenterology. (2019) 157:985–996.e2. doi: 10.1053/j.gastro.2019.06.003 31194979

[27] Standardization of Uveitis Nomenclature (SUN) Working Group. Development of classification criteria for the uveitides. Am J Ophthalmol. (2021) 228:96–105. doi: 10.1016/j.ajo.2021.03.061 33848532 PMC8526627

[28] Suh K Kyei I Hage DS. Approaches for the detection and analysis of antidrug antibodies to biopharmaceuticals: A review. J Separation Sci. (2022) 45:2077–92. doi: 10.1002/jssc.202200112 PMC9520450 35230731

[29] Sandborn WJ Wolf DC Kosutic G Parker G Schreiber S Lee SD. Effects of transient and persistent anti-drug antibodies to certolizumab pegol: longitudinal data from a 7-year study in crohn’s disease. Inflammatory Bowel Dis. (2017) 23:1047–56. doi: 10.1097/MIB.0000000000001100 28410341

[30] Vaughn BP. A practical guide to therapeutic drug monitoring of biologic medications for inflammatory bowel disease. J Clin Med. (2021) 10:4990. doi: 10.3390/jcm10214990 34768509 PMC8584740

[31] Cheifetz AS Abreu MT Afif W Cross RK Dubinsky MC Loftus EV. A comprehensive literature review and expert consensus statement on therapeutic drug monitoring of biologics in inflammatory bowel disease. Am J Gastroenterol. (2021) 116:2014–25. doi: 10.14309/ajg.0000000000001396 PMC9674375 34388143

[32] Argollo M Kotze PG Kakkadasam P D’Haens G. Optimizing biologic therapy in IBD: How essential is therapeutic drug monitoring? Nature Reviews. Gastroenterol Hepatol. (2020) 17:702–10. doi: 10.1038/s41575-020-0352-2 32879465

[33] Lee J Koreishi AF Zumpf KB Minkus CL Goldstein DA. Success of weekly adalimumab in refractory ocular inflammatory disease. Ophthalmology. (2020) 127:1431–3. doi: 10.1016/j.ophtha.2020.04.009 32423769

[34] Çam F Celiker H. Efficacy, retention rate and safety of adalimumab treatment in patients with non-infectious uveitis and scleritis: A real-world, retrospective, single-centre study. Eye (London England). (2024) 38:893–901. doi: 10.1038/s41433-023-02800-9 37884704 PMC10965934

[35] Liberman P Berkenstock MK Burkholder BM Chaon BC Thorne JE. Escalation to weekly adalimumab for the treatment of ocular inflammation. Ocular Immunol Inflammation. (2021) 29:1564–8. doi: 10.1080/09273948.2020.1749857 32407246

[36] Krieckaert CL Nurmohamed MT Wolbink GJ. Methotrexate reduces immunogenicity in adalimumab treated rheumatoid arthritis patients in a dose dependent manner. Ann Rheumatic Dis. (2012) 71:1914–5. doi: 10.1136/annrheumdis-2012-201544 22586169

[37] Kennedy NA Heap GA Green HD Hamilton B Bewshea C Walker GJ. Predictors of anti-TNF treatment failure in anti-TNF-naive patients with active luminal Crohn’s disease: A prospective, multicentre, cohort study. Lancet Gastroenterol Hepatol. (2019) 4:341–53. doi: 10.1016/S2468-1253(19)30012-3 30824404

